# Role of metal complexation on the solubility and enzymatic hydrolysis of phytate

**DOI:** 10.1371/journal.pone.0255787

**Published:** 2021-08-13

**Authors:** Mingjing Sun, Zhongqi He, Deb P. Jaisi

**Affiliations:** 1 Department of Plant and Soil Sciences, University of Delaware, Newark, DE, United States of America; 2 Department of Physical Sciences, Emporia State University, Emporia, KS, United States of America; 3 USDA-ARS Southern Regional Research Center, New Orleans, LA, United States of America; Government College University Faisalabad, PAKISTAN

## Abstract

Phytate is a dominant form of organic phosphorus (P) in the environment. Complexation and precipitation with polyvalent metal ions can stabilize phytate, thereby significantly hinder the hydrolysis by enzymes. Here, we studied the stability and hydrolyzability of environmentally relevant metal phytate complexes (Na, Ca, Mg, Cu, Zn, Al, Fe, Al/Fe, Mn, and Cd) under different pHs, presence of metal chelators, and thermal conditions. Our results show that the order of solubility of metal phytate complexes is as follows: i) for metal species: Na, Ca, Mg > Cu, Zn, Mn, Cd > Al, Fe, ii) under different pHs: pH 5.0 > pH 7.5), and iii) in the presence of chelators: EDTA> citric acid. Phytate-metal complexes are mostly resistant towards acid hydrolysis (except Al-phytate), and dry complexes are generally stable at high pressure and temperature under autoclave conditions (except Ca phytate). Inhibition of metal complex towards enzymatic hydrolysis by *Aspergillus niger* phytase was variable but found to be highest in Fe phytate complex. Strong chelating agents such as EDTA are insufficient for releasing metals from the complexes unless the reduction of metals (such as Fe) occurs first. The insights gained from this research are expected to contribute to the current understanding of the fate of phytate in the presence of various metals that are commonly present in agricultural soils.

## Introduction

Phosphorus (P) is an essential nutrient for all living organisms. Different forms of P (both inorganic and organic) in the environment have variable availability to plants and microorganisms. The inefficient use of P not only impacts agricultural productivity but also poses threats to water quality, such as eutrophication due to the potential loss of excess P from agro-ecosystem to water bodies. Thus, the characterization of P forms/species, availability, and potential impact are key factors for achieving the goals of modern sustainable agriculture by balancing the trade-off between agriculture and water quality.

Phytate consists of a cyclohexanehexol ring with six phosphate moieties linked via ester bonds. It makes up 50 to 80% of the total P in seeds [1–3] and accounts for the majority of total P in animal waste [4], especially for broiler litter and manure because of the presence of leftover or undigested seed phytate. Phytate is the dominant organic P form in soils [5, 6] due to plant residue and manure application. The phytate build-up in soils is often caused by the lack of enzymes for hydrolysis and its precipitation with metal ions [7]. The highly negative charge of phytate facilitates interaction with soil minerals and forms complexes with metals—the latter affects the solubility. In fact, stabilization of phytate in the environment is affected by the precipitation with metals in the following order: Cu(II) > Zn(II) > Ni(II) > Co(II),> Mn(II) > Fe(III) > Ca(II) [8, 9]. Precipitation, as well as direct sorption with metals or indirect sorption through polyvalent cations bridging, also decreases the bioavailability of phytate [6]. These edaphic factors together make phytate to be one of the most recalcitrant organic P compounds in the environment.

Plants can directly take up few inorganic P compounds. For phytate, the release of inorganic P (P_i_) from hydrolysis is required before plant uptake. The phosphate ester bond in phytate is quite stable under a range of physicochemical conditions [10]. For example, phytate is only partially hydrolyzed under thermal treatment with 2M hydrochloric acid at 140°C [11]. However, phytate can be hydrolyzed rapidly by phytate degrading enzymes, commonly known as phytase, which are produced by both plants and microorganisms [12–14]. It means, if favorable biogeochemical conditions exist in soils to support the production and expression of phytase activity, the hydrolysis of phytate can occur. However, due to the wide occurrence of organic matter and multiple metal ions in soils and agricultural waste such as manure, phytate often remains as sorbed or precipitated form, leading to its unavailability to phytase. Early investigation on the effects of cations on the hydrolysis of phytate found that the precipitation of phytate with Al and Fe inhibited the hydrolysis [15], but the hydrolysis was least impacted in the presence of Ca, Mg, and Mn metals [13].

Knowledge of the formation of metal phytate complexes and their fate is essential to develop a comprehensive understanding of the role of phytate in releasing P_i_ and support plant growth and minimize loss to open waters and thus to protect water quality. Because the released inorganic P (P_i_) from phytate after mineralization is readily available for plants and organisms, understanding the factors that affect phytate hydrolysis and phytate-phytase interaction in the environment is essential to determine the availability of phytate-sources of P_i_. However, limited studies have reported the stability and enzymatic hydrolysis of metal phytate complexes under different physicochemical conditions [13, 15]. The major aim of this study was two-fold: to (i) evaluate the stability and hydrolysis of metal phytate complexes under environmentally relevant pH, concentrations of chelators, and temperature, and (ii) investigate the inhibition of enzymatic hydrolysis of phytate due to metal complexation.

## Materials and methods

### Synthesis of metal phytate complexes

Commercial sodium phytate salt (Na_12_IP_6_, with purity > 90%) was purchased (from Sigma Aldrich) for this study. The other nine metal (Ca, Mg, Cu, Zn, Al, Fe, Mn, Cd, and mixed Al/Fe) phytate complexes were prepared using commercial sodium phytate salt and chloride form of each metal (Alfa Aesar) in the laboratory [16]. In this study, two types of Fe-phytate complex were prepared: i) Fe-1 phytate, prepared by adding NaOH into an acidified mixture of sodium-phytate and FeCl_3_, and ii) Fe-2 phytate, prepared by directly adding FeCl_3_ into sodium-phytate solution. The P: Fe ratio of the two complexes are different: Fe-1 phytate (1:1.21) and Fe-2 phytate (1:0.85). The representative metals chosen to be studied are based on: i) abundant metals in soils and manures (Na, Ca, Al, and Fe), ii) essential metals in living beings (Na, Ca, Fe, and Mg), iii) essential micronutrients for optimal growth and reproduction (Cu, Zn, and Mn), and iv) common soil contaminant (Cd).

Briefly, 30 mL of 0.05 M sodium phytate was mixed with different concentrations of metal chlorides. After adjusting the suitable pH for the metal phytate to precipitate, the product was then filtered and washed with deionized water to remove residual reactants. The precipitates were air-dried at room temperature and then oven-dried at 105°C for 1 h and stored in a desiccator at room temperature until use. All complexes were characterized by using Fourier transform infrared spectroscopy and solid-state ^31^P NMR spectroscopy and are reported in the previous study [16, 17]. The purity of these synthetic products, calculated based on the P content, ranged from 66 to 82%. The lower purity implies that some crystalline water or sorbed metal species might still be present in the product. The detailed information on synthesis, compositions of metal phytate complexes, and characterization are reported in a past publication [16].

### Analysis of the stability of metal-phytate complexes in different buffers

To test the stability of metal phytate complexes in different pH conditions and the presence of metal chelators (citric acid and ethylenediaminetetraacetic acid, EDTA), two sets of experiments in two different pHs were designed. The first set was carried out at pH 5.0 in 20 mM NaAc/HAc buffer. It included metal phytate complex (~3 mg in 10 mL buffer solution) i) without chelator, ii) with 3 mM citric acid, and iii) with 3 mM EDTA. The second set of experiments was identical to the first, except it was carried out at pH 7.5 using 20 mM Tris-HCl buffer: buffer without chelator, buffer + citric acid, buffer + EDTA. The initial concentration of metal-phytate complex in all experiments was ~300 mg/L (0.227–0.397 mM of phytate or equivalent of 1.36–2.38 mM phytate-P). All experiments were run in duplicates at room temperature (21 ± 2 ^o^C) for 90 days. At three selected time points (1 h, 30 d, and 90 d), 1 mL aliquot of homogenous solution/suspension was taken out and centrifuged for 10 min at 7000 rpm. Dissolved inorganic P (P_i_) in the supernatant was measured by the molybdate blue colorimetric method [18]. Total soluble P in the supernatant was determined after H_2_SO_4_-K_2_S_2_O_8_ digestion of 1/10 diluted solution. The difference between the total soluble P and P_i_ is defined as the soluble phytate P.

### Phytate determination by ion chromatography

To complement the colorimetric results, selected phytate metal complexes (at 30 d of incubation) were analyzed by ion chromatogram (IC) using a Dionex ICS-5000 equipped with an AS-AP autosampler, a DC thermal compartment, a SP pump, and a PDA detector. It included CarboPac PA-100 guard column (50 mm × 4 mm) and a CarboPac PA-100 analytical column (250 mm × 4 mm, 10 μm). The soluble fraction of phytate separated via centrifugation was injected in the IC column for quantitation. It was derivatized using Fe in perchloric acid (1 g/L Fe (NO_3_)_3_ in 0.33 M HClO_4_) first and quantified at 295 nm in the PDA detector, following our past method [19].

### Thermal stability of metal phytate complex

To test the stability of metal phytate complex at high temperature, one set of 3 mg dry powder of phytate-metal complex was dissolved in 10 mL of 20 mM NaAc/HAc (pH 5.0). It was centrifuged for 10 min at 7000 rpm to measure the inorganic P and total soluble P content before autoclave treatment. Another set of 3 mg dry powder of phytate-metal complex was autoclaved for 4 h (at the temperature of 121° C using saturated steam at the pressure of 15 psi). After the autoclave treatment, the sample was dissolved in 10 mL of 20 mM NaAc/HAc (pH 5.0) and centrifuged. The concentrations of inorganic P and total soluble P in the solution were measured, as explained above.

### Acid stability of metal phytate complex

To test the hydrolysis of metal phytate complexes under acidic conditions, 3 mg solid phytate complexes were suspended in 10 mL 1.0 M HCl. After 1 h and 1 d of reaction, aliquots were sampled and centrifuged. A 1 mL of supernatant was taken out to measure the concentration of dissolved P_i_ and total soluble P, as explained before. All experiments were performed in duplicate and at room temperature (21 ±2 ^o^C).

### Inhibition of enzymatic hydrolysis of phytate complexed with metals

To understand how the metal complexation impacts the enzymatic hydrolysis, selected metal-phytate complexes (2 mM P) were suspended in 5 mL of 100 mM glycine-HCl (pH 2.5) or 100 mM sodium acetate buffer (pH 5.0). A 3-phytase enzyme from *Aspergillus niger* (obtained from BASF) was added in each suspension at 0.1 U/mL. Metal-phytate complexes without the enzyme were used as controls. The reaction mixtures were incubated at an end-over-end shaker (40 rpm) at 21 (±2) ^o^C for 20 h. At the end of incubation, 0.1 mL aliquot was taken out and the concentration of soluble P_i_ was measured by a modified molybdate blue colorimetric method [20]. Total soluble P was determined after digestion and diluting to 1/10 of the aliquot, as before.

The effect of EDTA on the enzymatic hydrolysis of phytate complexed with Fe and Al was tested separately by adding 5 mM of EDTA in the incubation reaction. After the initial 20-h incubation in the presence of EDTA, released inorganic P and total soluble P in the solution were measured. Then sodium dithionite (10 mM) was added into the reaction mixtures and incubated for an additional 20 h to examine the effects of dithionite on hydrolysis. At the end of incubation, the concentration of soluble P_i_ and total soluble P were measured as above.

## Results and discussion

### Solubility and stability of metal phytate complexes in different buffers

**Fig 1:**
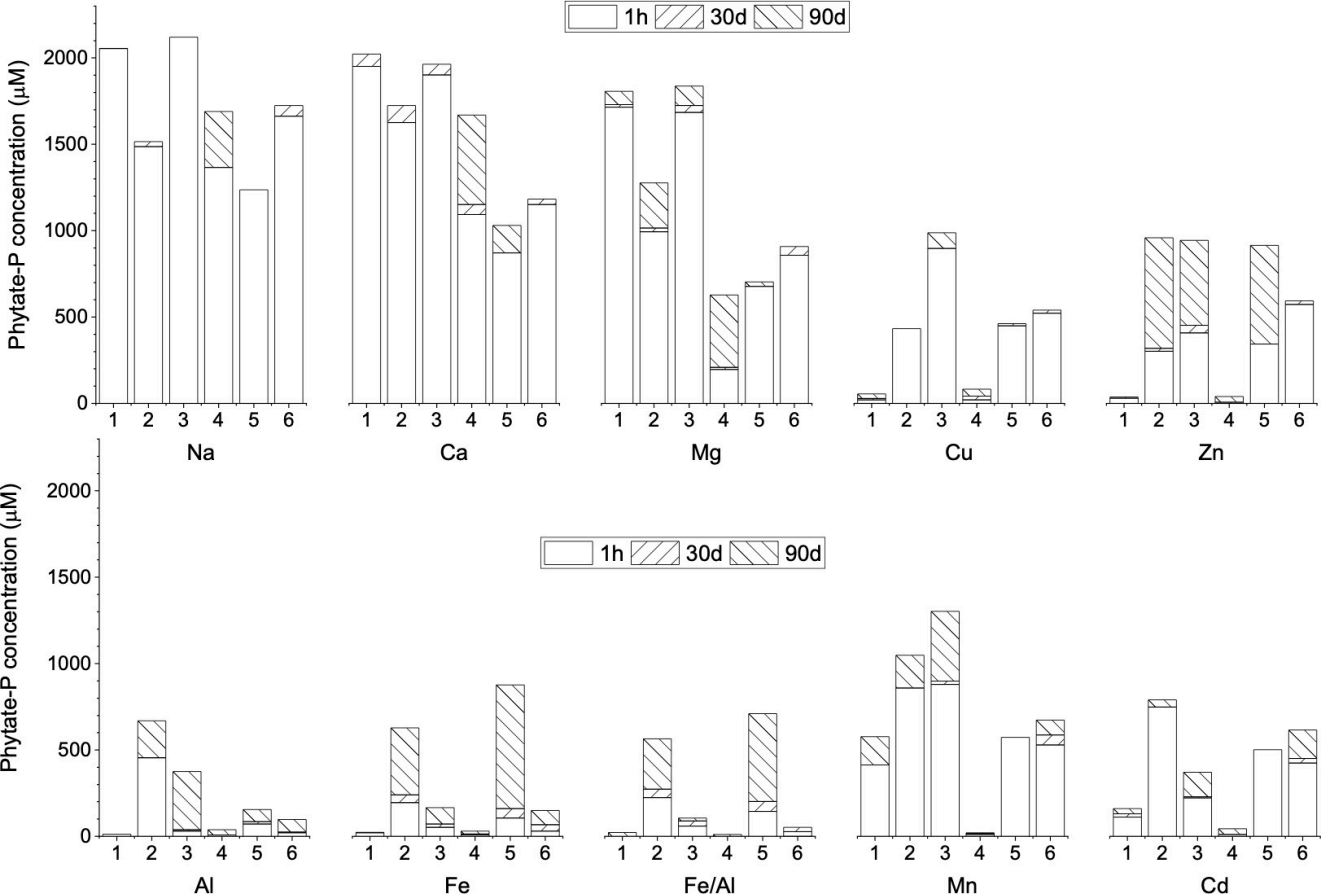
Dissolved concentration of metal phytate P in two different buffer systems at three different times (1h, 30d, and 90d). The initial concentration of metal phytate complex in the solution was 300 mg/L (about 0.227–0.397 mM of phytate or equivalent of 1.38–2.38 mM phytate-P) in each experiment. The concentration of metal phytate was quantified as the difference between total soluble P and inorganic P (P_i_). The numerals in X-axis represent: 1) 20 mM NaAc (pH 5.0), 2) 20 mM NaAc + 3 mM EDTA, 3) 20 mM NaAc + 3 mM citric acid, 4) 20 mM Tris-HCl (pH 7.5), 5) 20 mM Tris-HCl + 3 mM EDTA, and 6) 20 mM Tris-HCl + 3 mM citric acid.

The six phosphate moieties in phytate can have up to 12 coordination sites for complexation with cations. It forms monomeric and polymeric complexes or salts, which depend on the valency of coordinating cation and the nature of intermolecular bonding. Overall, the measured solubility in this study varied from 0 to 100% of phytate-P in different buffers and metal complexes (Fig 1). Average solubility of different metal phytate complexes after 1 h in NaAc buffer solutions was found to be in the following order: completely soluble (Na, 100%) > largely soluble [Ca (90.5%), Mg (64.5%)] > partially soluble [Mn (39.0%), Cd (8.23%), Cu (25.8%), Zn (14.3%)] > hardly soluble [Al (6.93%), Fe (4.31%) and Fe/Al mixture]. Comparison of the solubility among three buffer solutions showed that all metal phytate complexes were relatively more soluble (5 times for Al, two times for Fe, Mn, and Mg phytates) in slightly acidic condition (NaAc, pH 5.0) compared to near neutral pH (Tris-HCl, pH 7.5), but followed the same order. EDTA showed a strong chelating effect on all metals, which significantly improved the solubility. While the effect in Na and Ca phytates was insignificant, the solubility of other metal phytates was enhanced by 2–52 times. For example, adding EDTA on acetate buffer, concentration of soluble phytate in 1 h of treatment increased from 1.2% to 24.8% (Cu), 1.8% to 17.4% (Zn), 22.5% to 46.7% (Mn), 8.2% to 50.1% (Cd), 0.5% to 19.1% (Al), and 1.0% to 9.4% (Fe). It also increased the soluble metal phytate in 1 h incubation in neutral pH condition (Tris-HCl buffer), especially for Zn (0% in Tris-HCl while 19.9% in Tris-HCl + EDTA), Mn (0.6% in Tris-HCl while 31.2% in Tris-HCl + EDTA) and Cd (0.8% in Tris-HCl while 36.9% in Tris-HCl + EDTA). The enhanced solubility is caused by EDTA, a ligand with four carboxylate and two amine groups, chelating cations and freeing up phytate for the reaction [21]. Complexation of EDTA with phytate and inorganic P is used as a means to release them from soil matrices [22]. The stability of a cation–ligand complex, calculated to determine the competitive exchange between two specific ligands, has found that EDTA is an effective ligand, the chelating capacity of which depends on the solution pH, effective charge of the ligand, and inhibitory effect of polyvalent counterions in the solution [23].

Citric acid also enhanced the solubility of metal phytate complexes (by 2–49 times) when added to NaAc and Tris-HCl buffers. For example, in 1 h incubation, adding citric acid in Tris-HCl increased the concentration of soluble phytate-P from 8.6% to 37.8% (Mg), 1.1% to 29.8% (Cu), 0% to 33.1% (Zn), 0.5% to 28.8% (Mn), and 0.8% to 31.2% (Cd). However, citric acid was not as efficient as EDTA to enhance the solubility of Al and Fe phytates. For Al phytate, adding citric acid, soluble phytate increased from 0.5% to 1.27% (NaAc buffer), 0.3% to 0.9% (Tris-HCl buffer), while it increased from 0.5% to 19.1% (NaAc buffer), 0.3% to 3.0% (Tris-HCl buffer) when EDTA was added. For Fe phytate, adding citric acid, soluble phytate-P increased from 1.0% to 2.5% (NaAc buffer), 0.5% to 1.5% (Tris-HCl buffer), while it increased from 1.0% to 9.4% (NaAc buffer) and 0.5% to 5.1% (Tris-HCl buffer) when EDTA was present.

Long-term incubation almost always increased the solubility of metal phytate complexes. In 30 d incubation, solubility was relatively higher in the EDTA and citric acid buffers compared to those without chelators. For example, soluble phytate in 30 d of incubation in NaAc without chelators increased by 1.1, 1.2, and 1.0 times for Mg, Mn, and Cd phytates compared to 1 h incubation. But when the EDTA was present (NaAc+EDTA buffer), the increase was 2.0, 2.1, and 2.1 times for the three metal phytates, respectively. A higher increase in solubility is expected to be due to phytate complexation with EDTA and solubilization. Extending the incubation to 90 d resulted in further solubility. A 90 d incubation in NaAc + EDTA buffers resulted in the complete dissolution of Mg, Zn, Mn, and Cd phytates, while only 78% Cu, 30% Al, and 68% Fe phytates were soluble. The solubility of metal phytate complex is governed by a series of compounding and interdependent variables, including pH, temperature, ionic strength, and size and valency of the cation [24–28].

**Fig 2:**
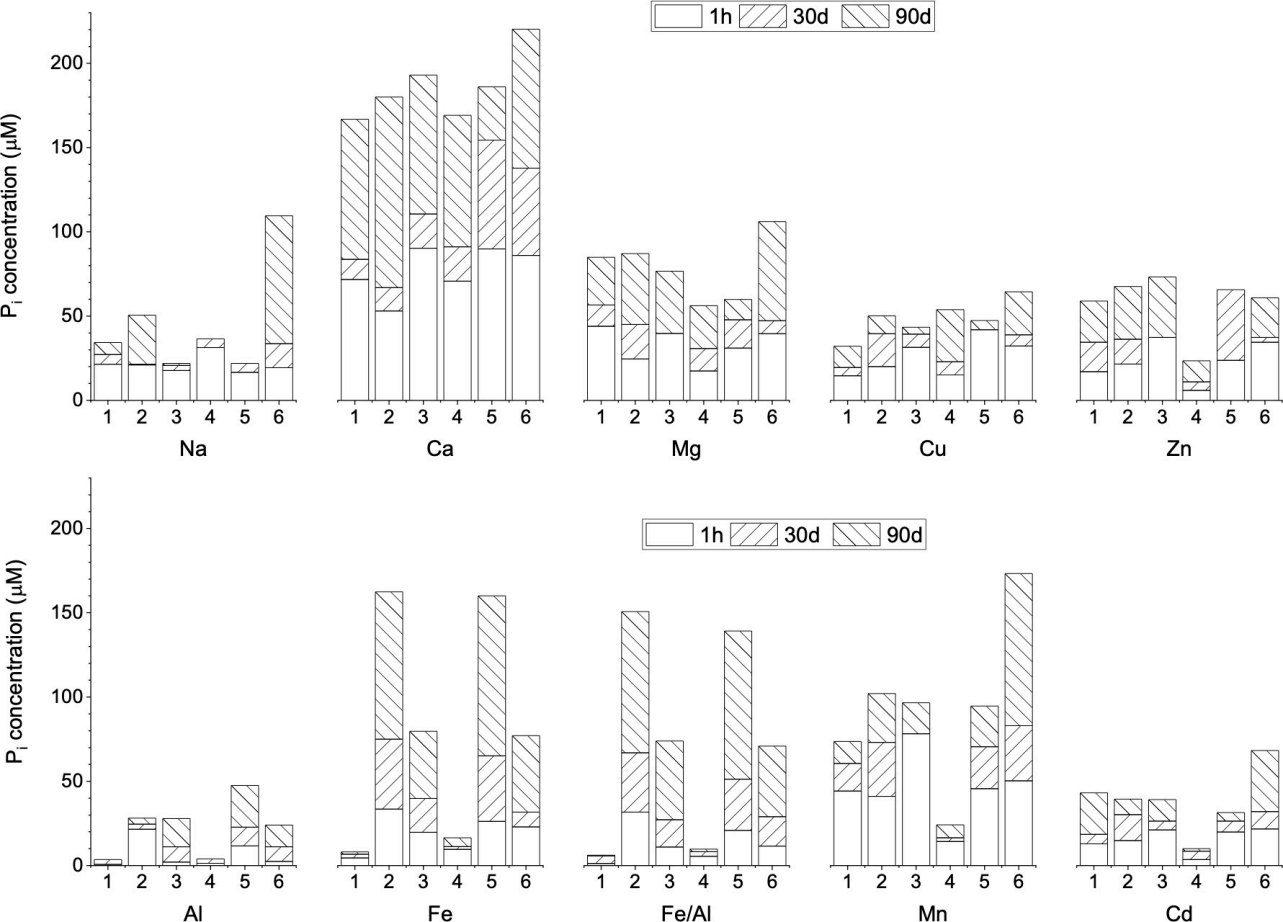
Dissolved inorganic P (P_i_) from metal phytate complexes. The initial concentration of metal phytate complex was 300 mg/L in each experiment (about 0.227–0.397 mM of phytate or equivalent of 1.38–2.38 mM phytate-P). The numerals in X-axis correspond to: 1) 20 mM NaAc (pH 5.0), 2) 20 mM NaAc + 3 mM EDTA, 3) 20 mM NaAc + 3 mM citric acid, 4) 20 mM Tris-HCl (pH 7.5), 5) 20 mM Tris-HCl + 3 mM EDTA, and 6) 20 mM Tris-HCl + 3 mM citric acid.

The concentration of dissolved P_i_ in the original solution (1 h incubation) was insignificant compared with total P (Fig 2), about 1% on average for most buffers and chelators, except Ca (~3.2% of total P) and Mn (~ 2.5% of total P). It varied significantly for different metal phytate complexes but was negligible compared to total dissolved phytate. However, the P_i_ concentration increased after long-term incubation. For example, on 30 d, less than 1.5% of phytate P was hydrolyzed and released inorganic P in the solution (0.3% of Na and Al phytates, 1.0% of Fe and Mn phytates, and 1.5% of Ca phytates). In 90 d incubation, inorganic P doubled for all metal complexes. About 1% of phytate was hydrolyzed for Na, Cu, and Al phytates, while more than 2% of phytate was hydrolyzed for Mg, Zn, Fe, and Mn phytates. However, for Ca phytate, the extent of hydrolysis was about 4.7–6.6% in 90 d in different buffers. Phytate is highly recalcitrant for abiotic hydrolysis [29] and self-hydrolysis is found to be insignificant. The commercial sodium phytate (extracted from rice) used in this study had a purity higher than 90% and is often reported to have a trace amount of phytase enzyme. Therefore, the measured concentration of P_i_ could be attributed due to the impurities in the phytate stock. It could also be caused by the limitation of measurement methods, which are discussed below.

### Comparison of phytate concentration by colorimetric and ion chromatography methods

**Fig 3:**
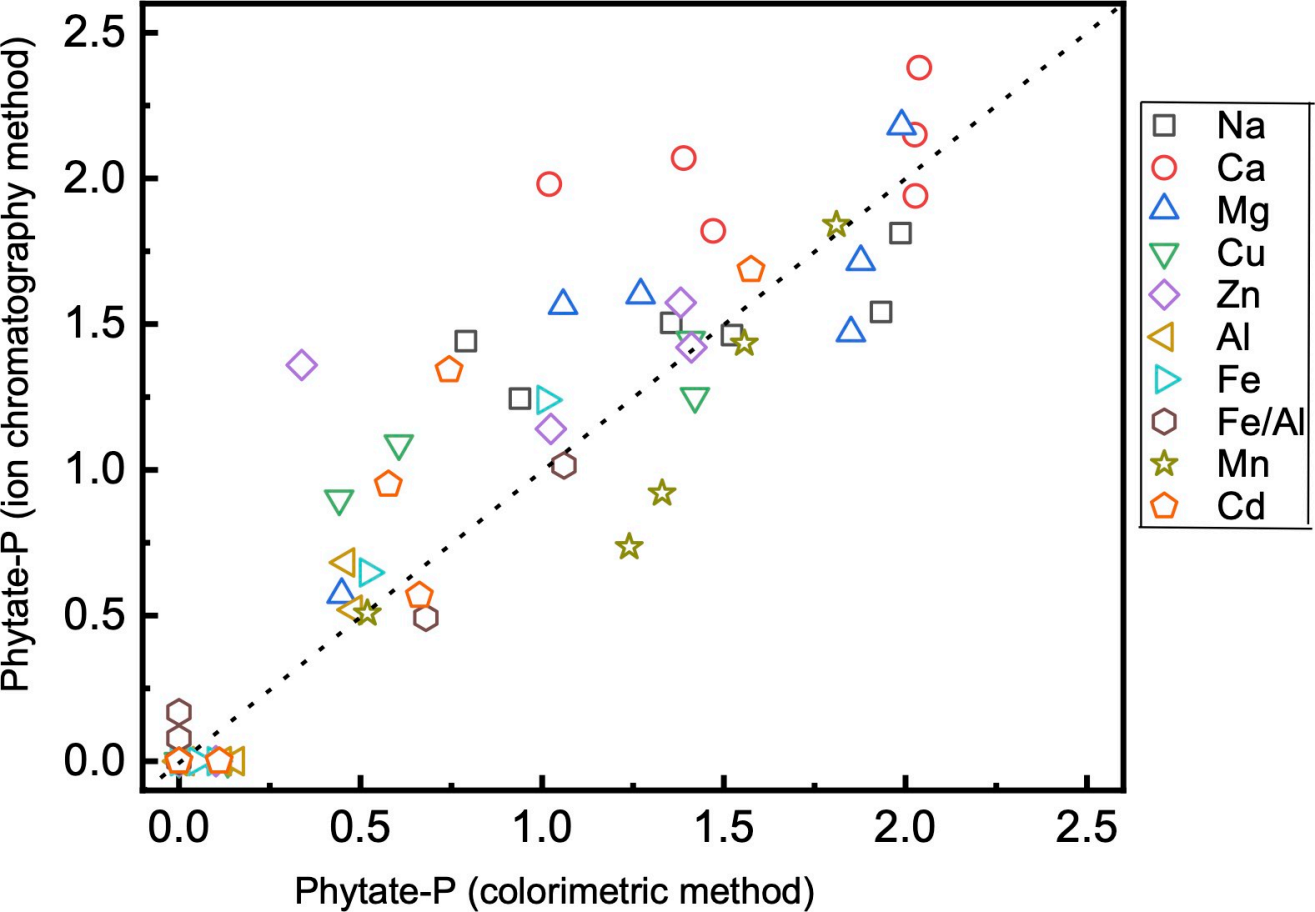
Comparison of dissolved phytate-P concentration at 30 d (in mM) from colorimetric and ion chromatography methods. For the colorimetric method, phytate-P was calculated as the difference between total soluble and inorganic P.

Comparison of solubility of phytate metal complexes (30 d) using colorimetric and IC methods showed a similar trend in different buffers (Fig 3). In most cases, measurements from IC are slightly higher than those from colorimetric methods. From the statistical analyses, the two methods are found not to be significantly different (at P < 0.05) on different metal species. The exception was Ca phytate, in which the concentration was about 30% higher in the IC method compared to the colorimetric method. This highlights the importance of selecting the appropriate method for measuring Ca-phytate: colorimetric method is good at low concentration because of the high sensitivity and IC at high concentrations due to less interference from metal ions in this method. On the role of buffers on impacting the measurement, results from the two methods are not statistically different (at P < 0.05) for all buffers except Tris-HCl + EDTA. The phytate concentration in the presence of Tris-HCl + EDTA measured in IC was about 80% higher than those using the colorimetric method, indicating Tris-HCl + EDTA strongly inhibits the colorimetric method of measurement.

The variable offset from 1:1 ratio between two methods could be attributed to factors including detection limits and interferences. One of the reasons is that the higher sensitivity of the colorimetric method, in which concentration can be measured at sub-micromolar level, while the IC was unable to detect phytate below 0.2 mM. However, the high sensitivity of the colorimetric method could also be compromised due to the interference of metal ions. For example, the colorimetric method is reported to likely overestimate the dissolved P in the presence of phytate [15]. In this study, phytate was measured indirectly by the colorimetric method after high-temperature hydrolysis. Unless phytate is completely digested to inorganic P, it could underestimate phytate-P content. Because the concentration of phytate is calculated by the difference from inorganic P determined before and after digestion, analytical error in the determination of inorganic P is high at low concentration [30]. Reagent-driven hydrolysis of many other organic P compounds commonly is a major cause of the overestimation of P_i_ [20], but phytate is very recalcitrant against all abiotic hydrolysis known so far. However, many metal ions (such as K, Na, Ca, Al, Mg, Fe, Zn, Fe, As, Si) and anions (NO_3_, Cl, HCO_3_) react with molybdenum and interfere with the colorimetric results [31–33]. While inhibiting concentration for most ions is usually higher than 1–10 mM, the synergistic effect of multiple ions in the solution to inhibit the colorimetric method is not quantified. Given that numerous ions are more likely to be present in the solution, the nature of interference in the colorimetric method can not be assessed.

The IC method of measurement of phytate concentration is indirect as well. Please note all phytate and partially degraded isomers were measured and quantified in IC following the past method [19]. The limit of detection is poor because phytate has no distinct chromophoric groups and cannot be detected in UV or visible light wavelengths. It is measured after post-column derivatization with Fe, which forms a Fe-phytate complex and then detected at UV wavelength (295 nm). The sensitivity of Fe-phytate complex was lower in the IC than in the colorimetric method. Overall, the colorimetric method has a lower limit of detection than IC but could be easily affected by interference from metals in the solution. The ion chromatography has a poor limit of detection but can be more robust for measuring higher concentrations of phytate complexes.

### Stability of metal phytate complexes at high temperature and low pH

**Fig 4:**
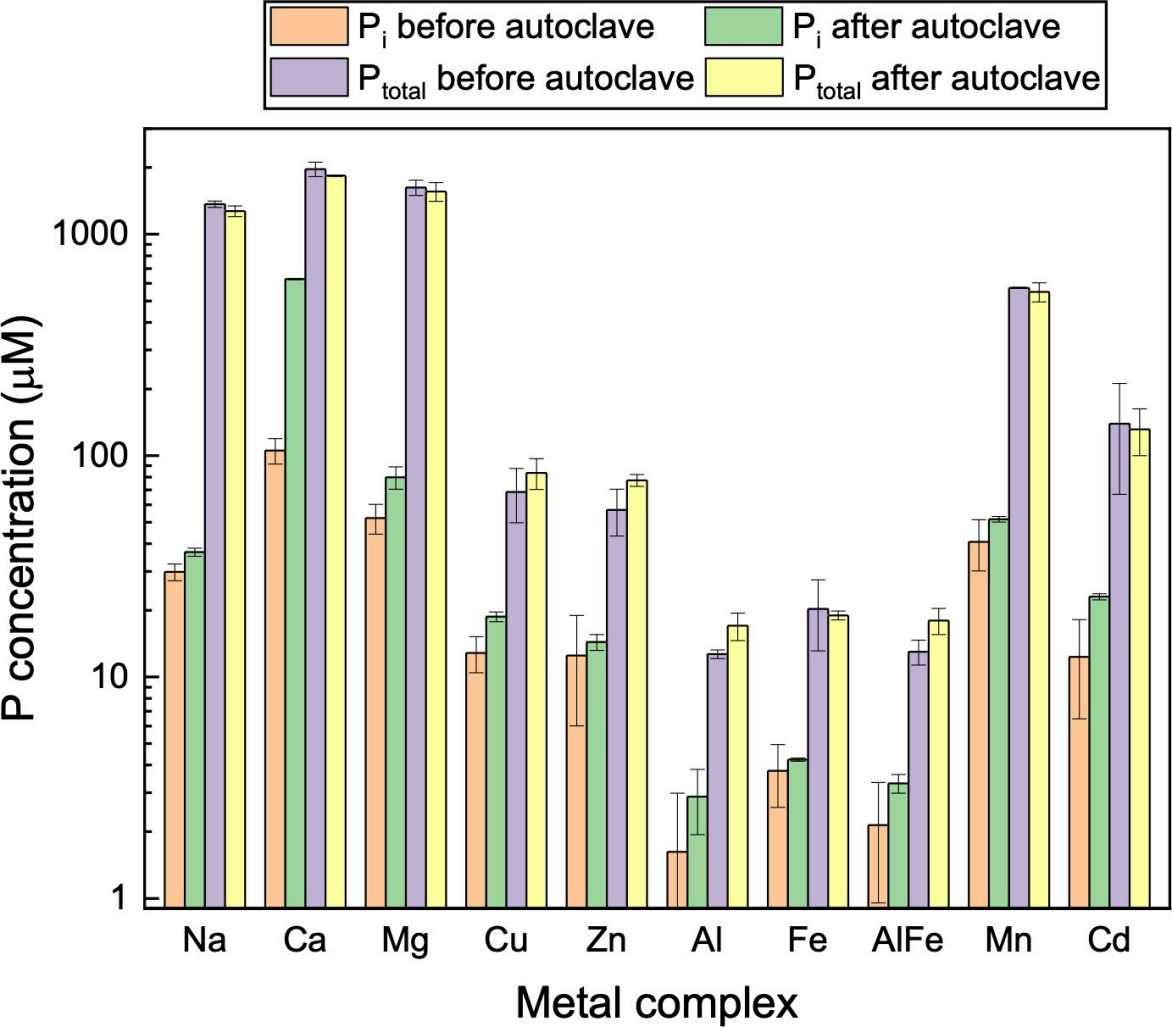
Thermal stability of anhydrous metal phytate complexes in autoclave treatment (at 121° C and 15 psi). The initial concentration of metal phytate complex was 300 mg/L (about 0.227–0.397 mM of phytate or equivalent of 1.38–2.38 mM phytate-P) in each experiment. Different P measurements and calculations are the same as in Fig 3.

The results on the thermal stability of different dry metal phytate complexes are shown in Fig 4. A negligible difference was found both in inorganic and total soluble P before and after autoclave in most complexes, suggesting phytate is resistant to high temperature irrespective of the type of metal complexation. The extent of decomposition of metal phytate was less than 0.05% for Al and Fe phytates, less than 1% for Cu, Zn, Mg, and Cd phytates, while less than 1.6% for Na and Mg phytates. The exception, however, is Ca phytate, in which the soluble inorganic P increased from 105 to 666 μM, indicating about 28% phytate was decomposed during autoclave treatment. The high-temperature treatment in this study was performed in fully anhydrous powders and pure phytates are reported to be fairly stable to heat [34]. However, dissolved phytate can be hydrolyzed at high temperatures [10] and the metal phytates are relatively less hydrolyzed than pure phytate [35].

**Fig 5:**
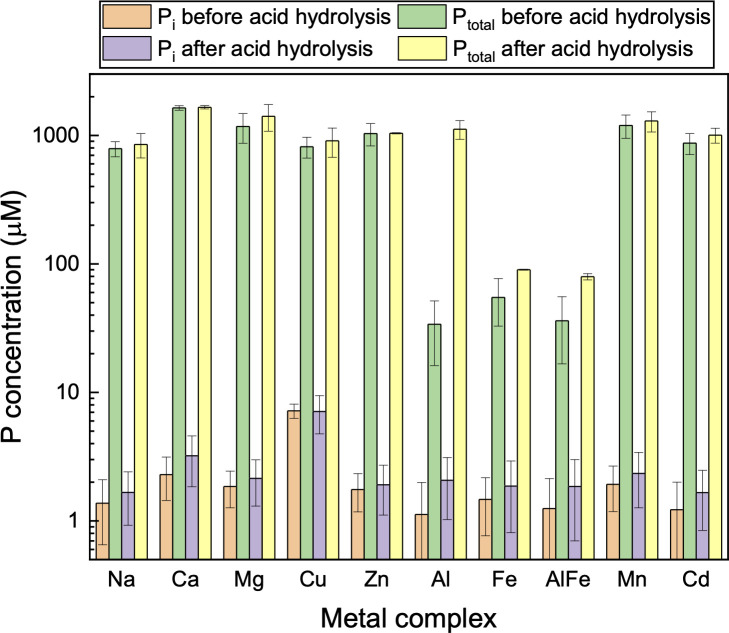
Stability of metal phytate complexes in acid (1 M HCl). P_i_ and P_total_ before acid hydrolysis refer to that already present in the solution before treatment. The initial concentration of metal phytate complex in the solution was 300 mg/L in each experiment. The difference of soluble phytate P before and after hydrolysis is insignificant.

Results on acid stability experiments on phytate metal complexes are shown in Fig 5. No or insignificant increase of inorganic P (less than 0.05% of phytate P) from 1 h to 1 d showed all the metal phytate complexes were stable in 1 M HCl. The order of solubility of different complexes is: Na, Ca, Mg > Mn, Cd > Cu, Zn > Al and Fe phytates. No increase in total P showed there was no enhancement of solubility after incubating for a longer time. Interestingly, the concentration of soluble Al phytate increased 34 times (from 34 μM to 1117 μM) after 1 M HCl treatment for 1 d. This result has implications for using HCl to extract Al phytate from its mixture with Fe phytate since Al and Fe are commonly associated P in agricultural wastes and removed simultaneously using the traditional extraction method [36].

### Enzymatic hydrolysis of metal phytate complex

**Fig 6:**
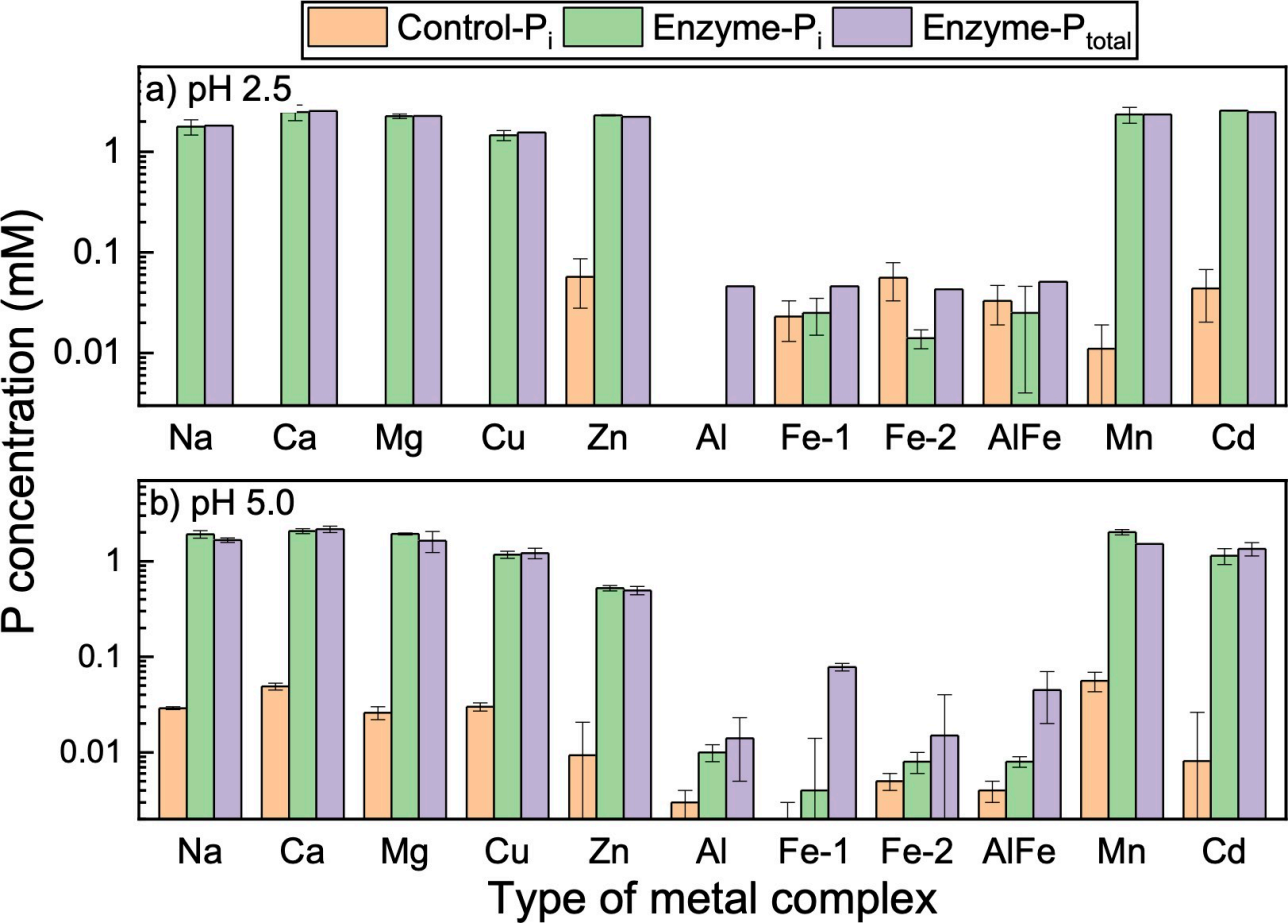
Released inorganic P in phytate metal complexes (2 mM P) after 20 h incubation with Aspergillus enzyme at pH 2.5 and 5.0. Control refers to treatment without the enzyme. For enzymatic treatments, 0.1 U/mL Aspergillus phytase was used.

None or negligible concentration (< 0.1 mM) of soluble inorganic P was detected in controls of all phytate complexes (Fig 6). It means that these complexes are stable during the incubation period. In the presence of *Aspergillus* phytase (a 3-phytase enzyme), complete hydrolysis of Na phytate complex occurred both at pH 2.5 and 5.0. For other complexes, different degree of hydrolysis was observed in two pHs. No significant enzymatic hydrolysis was observed in Fe, Al, and Al/Fe phytate complexes. Apparently, increased bonding in metal (III) ions and phytate limited the solubility of these metal complexes, leading to the inaccessibility of the enzyme to attack the P-O bond. These observations are consistent with previous reports that Al and Fe ions inhibit hydrolysis of soluble phytate by forming insoluble metal phytate complexes [15, 37].

Near-complete hydrolysis of Ca, Mn, Mg, Zn, and Cd phytate complexes occurred at pH 2.5, but was partial at pH 5.0 (83–84% of Ca, Mn and Mg phytate, 23% of Zn phytate, 44% of Cd phytate). About 73% Cu-phytate was hydrolyzed under pH 2.5 while about 59% under pH 5.4. The *Aspergilus niger* enzyme has two pH optima: 5 to 5.5 (100%) and 2.0 to 2.5 (60%) [38]. However, the efficacy is significantly impacted by various substitutions made in the enzyme to maximize hydrolysis in animal gut conditions. For example, substituting amino acids in the substrate-binding site changes enzyme relative activity to be almost the same at pHs 2.5 and 5.5 but about 50% higher at pH 3.5 [39]. Among different metals, a higher chelation effect of trivalent metal ions than bivalent with phytate limits the solubility of these metal complexes, leading to inaccessibility for the enzymatic attack on the P-O bond. The sequestering ability of phytate toward the metal cations increases with increasing pH [40], lower pH favors the solubility of these ions [41]. It is reported that phytate hydrolysis by a 3-phytase in vitro at pH 2.5 and 6.5 is reduced in the presence of Ca and the effect is greater at pH 6.5 [42], due to the high binding affinity of Ca-phytate [43]. Similarly, Cu influences phytate hydrolysis differently at different pHs: no inhibition at pH 2.5, while Cu addition greatly inhibited the hydrolysis at pH 5.5 and pH 6.5 [44]. Results from this study are also consistent with previous reports that Al and Fe ions inhibit the hydrolysis of soluble phytate by forming insoluble metal phytate complexes [30].

**Fig 7:**
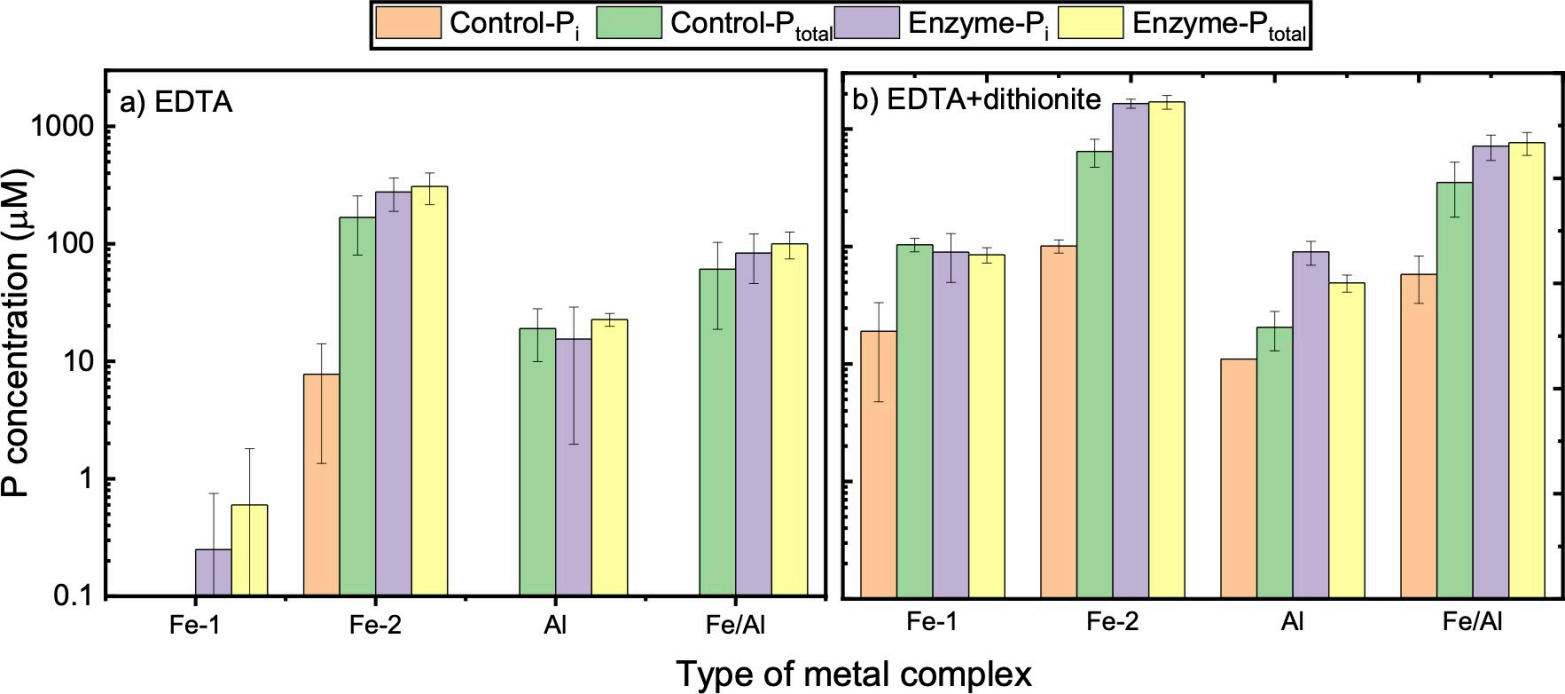
Effect of EDTA and dithionite on the dissociation of metal-phytate complexes (starting concentration of 2 mM P) at pH 5.0. (a) Incubation for 20 h in the presence of EDTA (5 mM); (b) Incubation for an additional 20 h after spiking with sodium dithionite (10 mM). Aspergillus phytase enzyme (0.1 U/mL) was used for the hydrolysis.

Inclusion of EDTA in the enzyme and metal-phytate incubation did not significantly increase the solubilization or dephosphorylation of Fe- and Al-phytate complexes (Fig 7), and change was limited 5 to 15%. EDTA has been reported to remove the inhibitory effect of Fe (III) and Al (III) ions on hydrolysis of phytate by chelating metal ions, which otherwise interact and precipitate phytate [23]. The fact that EDTA exerted negligible to limited effects on solubilization and hydrolysis of metal (III) phytate complexes indicates that EDTA is less effective to competitively chelate metal (III) ions bound previously to phytate. It is, however, important to highlight that the ability of EDTA to decouple metal complexed phytate allowing enzyme binding and hydrolysis to occur depends on type and concentration of inhibiting ions and molar ratios with the phytate complex. For example, adding EDTA ligand to a mixture of phytate and Al (conc. > 0.75 mM) is found to reverse the inhibitory effect of metal ions on the hydrolysis reaction. The ability of EDTA to decouple counterion and thus facilitate phytase to hydrolyze phytate is pronounced at pH 4.5 when EDTA is present primarily as trivalent (HEDTA^3−^) ion. When the pH is increased to 6.0, the ability of EDTA to reverse the inhibitory effects is not effective [23].

Further incubation of the metal complexes with dithionite did not markedly enhance the release of P from Fe-1 and Al phytate complexes but significantly enhanced the solubility (by 33%) and hydrolyzability (by 83%) of Fe-2 phytate. These two factors for Fe/Al phytate were 18% and 36%, respectively (Fig 7). These observations demonstrate that dithionite is a more efficient reagent to solubilize Fe (III) phytate than EDTA. Dithionite, a strong reductant, reduces Fe (III) to Fe (II), thus enhances the solubility of Fe-related P complexes [45]. The enzyme, however, seemed to act not only on solubilized phytate but also on precipitated metal phytate complex, albeit the efficiency is significantly limited, as shown by the increase in P in the presence of enzyme than with control (Fig 7). Fe-2 phytate was more soluble and hydrolyzable than Fe-1 phytate in the presence of EDTA and EDTA/dithionite. FTIR spectra, however, indicated no significant differences between Fe-1 and Fe-2 phytate complexes [16]. We presume that the difference is attributed to the high metal content in Fe-1. The measured P/Fe molar ratio is 1:1.21 in Fe-1 phytate and 1: 0.85 in Fe-2 phytate. The P/Fe ratio in Fe-1 phytate complex was 81% higher than stoichiometry, indicating the presence of impurities such as sorbed Fe or precipitates as Fe (oxy) hydroxide. Further investigation of the role of complexed and free metals and related chemistry may shed light on the changes in interaction and transformation of metal-phytate species. For instance, Ca and Mg are reported to limit the solubility of poultry litter P [46, 47], and Al and Fe containing chemicals or byproducts have been proposed to stabilize P in manure, thereby decrease the P in the runoff [48–50]. On the other hand, phytate content in foods impairs the bioavailability of minerals [28, 51]. For example, in vitro availability of Fe and Zn in whole pearl millet flour was significantly improved by phytate degradation [52]. These results highlight that the phytate complexes such as those used in this study are influenced by various factors, including optimal pH of enzyme, concentration, composition, and relative ratios of chelators and metals.

In summary, the reactivity of phytate and the inhibitory effects of metal ions on the hydrolysis by phytase significantly affect the fate of phytate and other inositol phosphates. It should be pointed out that advanced spectroscopic techniques have shown different conformational structures of phytate complexed with different metals with particular distinction among mono- and multi-valent ions [53–55]. Future research on developing the relationship of conformational structure to hydrolysis and impact of released metal will help develop the mechanistic relationship and provide insights on the availability of phytate in soil and other environments.

## Conclusions and implications

The reactivity of phytate and the inhibition towards hydrolysis by phytase caused by complexation with metal ions significantly affect the fate of phytate and other inositol phosphates in the environment. In this study, the roles of various factors affecting the solubility and hydrolysis of dissolved and insoluble metal phytate complexes were studied. The solubility of ten metal phytate complex are found as follows: i) under complexation with metals: Na, Ca, Mg > Cu, Zn, Mn, Cd > Al, Fe; ii) at different pHs: NaAc (pH 5.0) > Tris-HCl (pH 7.5); and ii) under chelation: EDTA> citric acid. The stability study showed that most metal phytate complexes were stable in different buffer solutions (up to 90 d) except Ca phytate, which hydrolyzed 4.7–6.6%. The thermal stability study showed that all dry metal phytate complexes were quite stable. Ca phytate was the least stable and was hydrolyzed 28% under autoclave treatment. The stability in the presence of strong acid was high for all metal phytate complexes, except Al phytate, the solubility of which increased 34 times during 24 h treatment in acidic solution. *Aspergillus* phytase was generally efficient in hydrolyzing phytate complexed with different metals. For example, near-complete hydrolysis of Ca, Mn, Mg, Zn, and Cd phytate complexes occurred at pH 2.5, while hydrolysis was partial at pH 5.0 (83–84% of Ca, Mn and Mg phytate, 23% of Zn phytate, 44% of Cd phytate). About 73% Cu-phytate was hydrolyzed under pH 2.5 while about 59% under pH 5.4. However, the limited P_i_ was released from enzymatic hydrolysis of Fe and Al phytate complexes. EDTA, which has been used to relieve the inhibition of Fe(III) and/or Al(III) ions on enzymatic hydrolysis of soluble phytate, was not quite efficient to facilitate the enzymatic release of P from metal (III) phytate complexes. While dithionite dissolved 33% of Fe-2 phytate and 83% hydrolyzed, its effect was limited in Fe/Al phytate (dissolving 18% and hydrolyzed 36%). Overall, our results highlight the contrasts on solubility, hydrolyzability, and thermal stability found in this study among diverse metal phytate complexes that are likely to form in soils and waters, and inside animal bodies or in wastes. These results provide useful information to better understand the fate and bioavailability of phytate in the environment.
